# δ^15^N and δ^13^C cycles in narwhal (*Monodon monoceros*) embedded teeth reveal seasonal variation in ecology and/or physiology

**DOI:** 10.1098/rsos.242237

**Published:** 2025-03-12

**Authors:** Shu-Ting Zhao, Cory J. D. Matthews, Cortney A. Watt

**Affiliations:** ^1^ Arctic Aquatic Research Division, Fisheries and Oceans Canada, Winnipeg, Manitoba, Canada; ^2^ Department of Biological Sciences, University of Manitoba, Winnipeg, Manitoba, Canada

**Keywords:** narwhal, stable isotopes, teeth, GLGs, accessory layers, seasonality

## Abstract

Monitoring Arctic marine mammals in response to rapid climate change requires reliable longitudinal data. To obtain such data is challenging, but sequential measurements of stable isotopes (SI) from metabolically inert tissues like dentine allow for chronological reconstruction of SI data that can provide insights into whale life history, behaviour and physiology. This study examined dentine samples from narwhal embedded canines to reconstruct individual SI profiles and assess intra-annual variation in δ^15^N and δ^13^C. The individual δ^15^N and δ^13^C profiles of all 31 narwhals exhibited cyclical oscillations. The majority of δ^15^N and δ^13^C oscillations (>50%) occurred within the annual growth layer groups (GLGs), suggesting seasonal variation. The mean magnitude of SI oscillations per individual ranged from 0.4‰ to 2.5‰ for δ^15^N and 0.2‰ to 1.1‰ for δ^13^C. Such intra-annual SI oscillations may reflect variability in narwhal ecology related to environmental variation (e.g. seasonal changes in baseline SI and diet associated with narwhal migration) and/or changes in narwhal physiology (e.g. seasonal energetics), highlighting the utility of SI profiles for long-term monitoring of narwhal’s ecological and physiological responses to a changing Arctic.

## 1. Introduction

Arctic mammals have evolved unique physiological [1,2], morphological [3,4] and behavioural adaptations [5,6] to survive extreme seasonal and inter-annual fluctuations in their environment; however, these long-living and slow-growing animals are typically vulnerable to rapid unidirectional changes [6]. Climate warming has resulted in reduced Arctic sea-ice with pronounced intra- and inter-annual variability over the past 30 years [7], which has led to altered nutrient cycling, shifts in phytoplankton community composition [8,9], and subsequent shifts in growth, abundance and distribution of Arctic fish species [10,11]. Ongoing environmental changes have been observed and predicted to impact Arctic marine mammals, including eliciting changes in their timing of migration, habitat use and feeding patterns [12–14].

**Figure 1.**
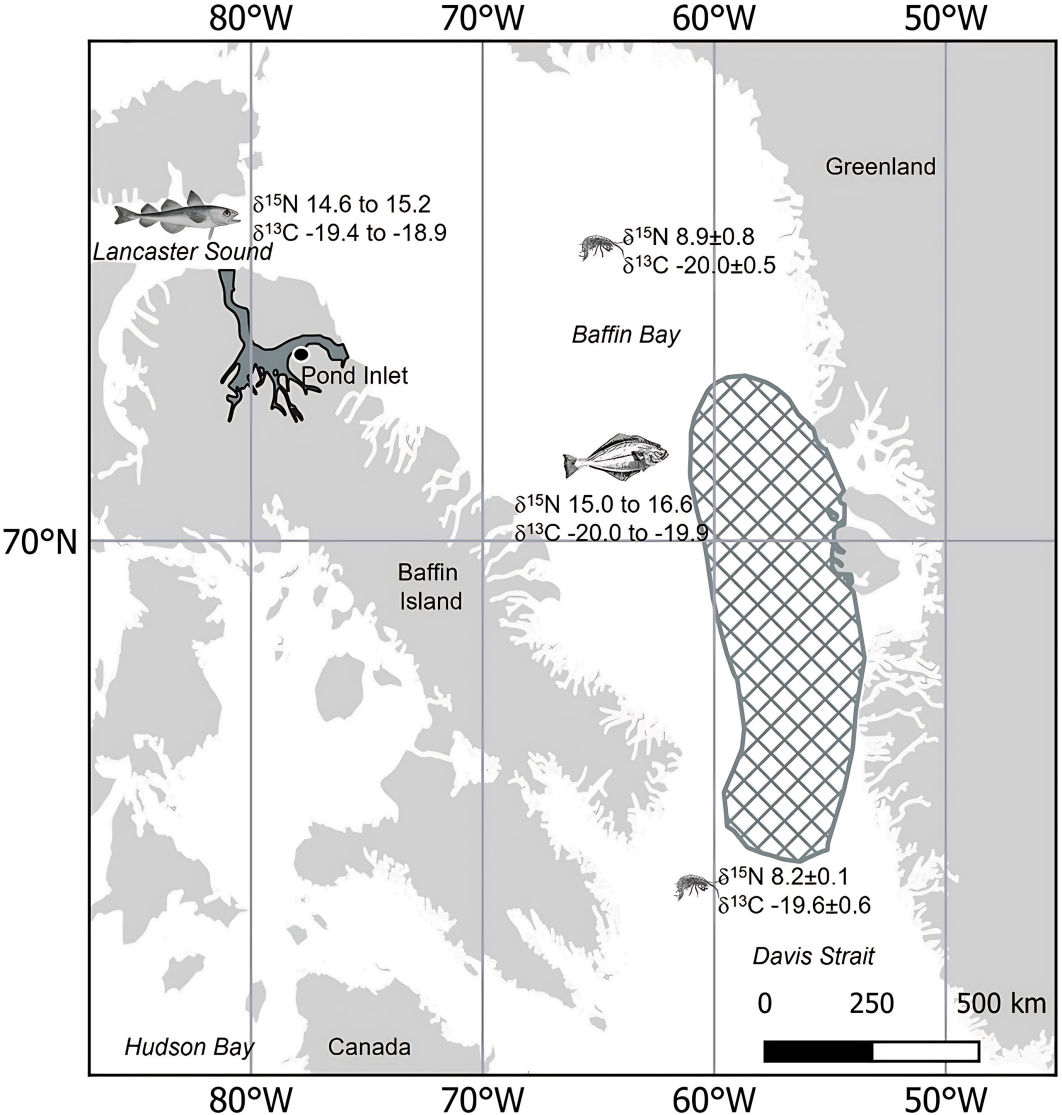
Map of Baffin Island (Nunavut, Canada), where 31 narwhals were harvested near Pond Inlet in 1982, 1983 and 2017 during subsistence hunting and in 2015 during a humane harvest. Baffin Bay narwhals summer in the northern inlets of Baffin Island and overwinter in central and southern Baffin Bay and Davis Strait [15,16]. There is a slight baseline gradient such that δ^15^N of zooplankton (e.g. *Themisto libellula*) declines from north to south, with an inverse trend in δ^13^C (grid area) [17]. Prey values also differ in δ^15^N and δ^13^C across the regions, where narwhal are eating primarily Arctic cod in the northern summering grounds and Greenland halibut in the wintering area [18,19].

The world’s largest narwhal (*Monodon monoceros*) population, the Baffin Bay population, summers along the northern inlets of Baffin Island and within the ice-free waters of the Canadian Archipelago, before migrating to central and southern Baffin Bay and Davis Strait for the winter [15,16] (figure 1). Stomach content analysis suggests that Baffin Bay narwhals forage intensively during winter on Greenland halibut (*Reinhardtius hippoglossoides*) and squid (*Gonatus fabricii*), and forage more opportunistically during summer, primarily on Arctic cod (*Boregadus saida*) [18,19]. Stable isotope (SI) analysis has also indicated seasonal changes related to narwhal diet which was hypothesized to be associated with their migration between summer and winter grounds [20]. However, changes in narwhal foraging behaviour, habitat use, migration phenology and energetics are expected due to alterations in prey composition and abundance, declining sea ice, increased vessel traffic and increased killer whale presence in narwhal summering areas [14,21–23]. Longitudinal datasets with intra-annual resolution are required for monitoring and assessing narwhal ecological and physiological variability.

Consumer SI ratios of nitrogen (δ^15^N) and carbon (δ^13^C) are driven by the SI composition of their prey, their own physiology and the baseline SI imparted by biogeochemical processes at the base of the food web [24,25]. The SI of metabolically active tissues, such as skin and muscle, can provide insights into an animal’s past ecology over relatively short periods (e.g. months to a year), reflecting changes in diet, habitat use and other ecological factors based on the turnover rates of these tissues [26]. Teeth, on the other hand, are metabolically inert structures that grow continuously and remain relatively unchanged once formed [26]. The teeth of many whales are characterized by the deposition of dentine as annual growth layer groups (GLGs) [27,28]. Sequential measurement of SI in annual GLGs along the axis of tooth growth has allowed for chronological reconstruction of whale movements [29], diet [30] and ontogenetic development [31] that have been otherwise unattainable.

**Figure 2.**
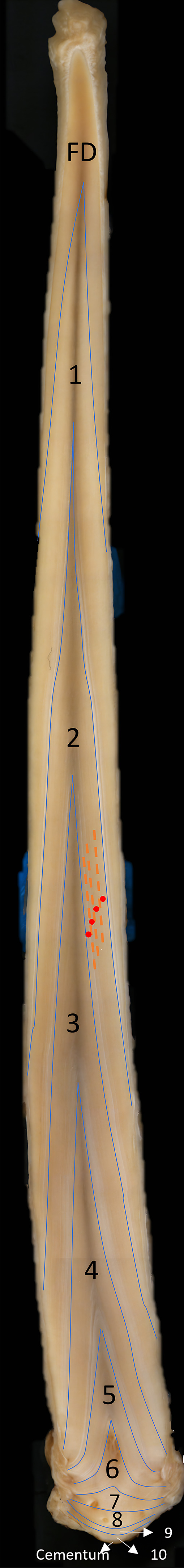
Cross-sectioned embedded tooth of narwhal specimen PI-83-11. Dentine growth layer groups (GLGs 1−10) are marked as layers bounded by blue solid lines. Foetal dentine (FD) is a layer deposited during gestation. This tooth is occluded by cementum after GLG 10. Four accessory layers in GLG 2 are indicated by red dots separated by orange dashed lines.

Narwhals have two canine teeth that develop *in utero* and continue to deposit dentine annually postpartum [32,33]. Dentine GLGs in narwhal canines have multiple accessory layers that presumably reflect intra-annual growth patterns [34,35] (figure 2). The relatively large GLGs in embedded canines facilitate the sampling of intra-annual accessory layers, thus overcoming a typical limitation of tooth studies constrained to whole-GLG sampling by small tooth size, thereby providing only annual resolution [30,36]. In this study, we sampled accessory layers within dentine GLGs of narwhal embedded canines to analyse δ^15^N and δ^13^C profiles with intra-annual resolution. Seasonal variations in both measures were interpreted within the context of potential ecological (seasonal diet shifts and migrations) and physiological (e.g. breeding status, nutritional stress, growth rate) factors that may inform narwhal responses to future environmental changes.

## 2. Methods

Embedded teeth with well-defined GLGs from 31 narwhals (females (*n* = 17) and males (*n* = 14)) harvested by Inuit hunters near Pond Inlet, Nunavut, Canada (figure 1), were selected for this study. Full descriptions of tooth extraction and preparation are available in Watt *et al*. [33] and Zhao *et al*. [31]. A dentine GLG typically comprises a wide light layer and a narrow dark layer with variable numbers of accessory layers within (figure 2); the age of an animal can be estimated by counting dentine GLGs [31,32]. However, dentine GLGs in narwhal embedded teeth become compressed and eventually occluded by cementum (often at GLG 8−16) (e.g. figure 2), which makes estimating absolute age and calendar year of formation impossible for older whales.

Dentine was collected from individual accessory layers of longitudinally sectioned teeth (figure 2) using a micromill fitted with a 300 μm drill bit. The accessory layers were micromilled up to GLG 11, beyond which compressed growth prevented discrete sampling of accessory layers. Sampling of accessory layers yielded limited material for analysis. To avoid sample loss during the acidification process typically used to remove inorganic carbon, bulk dentine samples (0.7 ± 0.02 mg) were analysed for δ^15^N and δ^13^C following studies that have shown that δ^13^C values of bulk and acidified dentine did not differ significantly in toothed whales (e.g. [37,38]). A total of 839 dentine accessory layer samples were analysed at Fisheries and Oceans Canada, Winnipeg, MB, using an Elemental Combustion System CHNS-O (ECS 4010, Costech Analytical Technologies Inc., USA) connected to a DELTA-V mass spectrometer (ThermoFisher Scientific, Bremen, Germany). Isotope values are reported as heavy to light isotopic ratios relative to international standards. The standard deviation (*SD*) of repeated measurements (*n* = 390) of certified standards (USGS 40 and 41 a, L-glutamic acid) was 0.1‰ for δ^15^N and 0.1‰ for δ^13^C, while the *SD* for sample duplicates (*n* = 124) was 0.1‰ and 0.1‰ for δ^15^N and δ^13^C, respectively. The atomic C:N ratio of bulk dentine was 3.5 ± 0.1 (*SD*).

Variable numbers of accessory layers sampled in each GLG prevented analysis of isotope profiles using time series analysis, which assumes stationarity over constant time increments [39]. Instead, low frequency variation related to ontogeny (e.g. weaning-associated δ^15^N decline, 30) or other factors (e.g. temporal baseline shifts) was removed from the isotopic profiles of individual narwhals using a Gaussian low pass filter (e.g. [40,41]), and the remaining high frequency (i.e. seasonal) oscillations were assessed visually. An oscillation was defined as a change from peak-to-trough or trough-to-peak regardless of the GLG patterns. The peak-to-trough difference (magnitude) was calculated by subtracting the trough value from the peak value of each oscillation. The mean δ^15^N and δ^13^C magnitudes for each whale were averaged from the magnitudes across the isotopic profiles of the individual narwhals. The range of magnitudes, and the mean magnitude of each narwhal SI profile were qualitatively assessed. Data were detrended and visualized using R software [42].

## 3. Results

**Figure 3.**
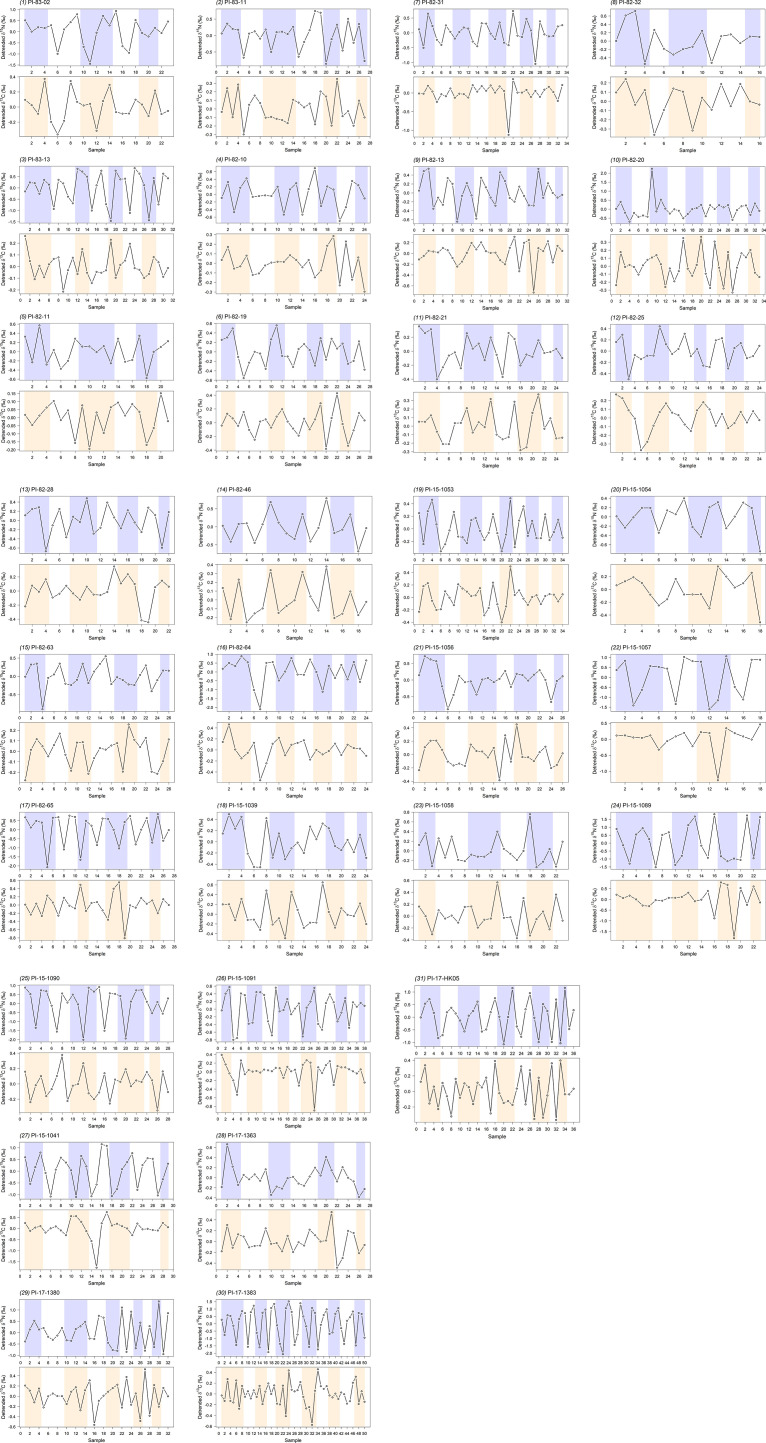
Detrended δ^15^N and δ^13^C profiles of dentine sequentially sampled from accessory layers in growth layer groups (GLGs) in embedded canines of 31 narwhals show oscillation(s) within years. Sample numbers are indicated along the *x*-axis with individual GLGs highlighted using alternating colour bands.

The number of accessory layers per GLG ranged from 2 to 8 with a mean of 3.6. Early GLGs (GLG 1−5) tended to contain more accessory layers (≥ 4) than later GLGs (GLG 6−11; ≤ 3). There was considerable variation in δ^15^N and δ^13^C across GLGs in all whales (*n* = 31) (figure 3) with all whale profiles showing obvious or some cyclic patterns for both δ^15^N and δ^13^C. Although there did not seem to be a perfect match between the cyclic oscillations and GLG patterns, more than half of the oscillations in each SI profile repeated approximately every year (figure 3). In most cases, there was at least one oscillation (one peak-to-trough or one trough-to-peak) per GLG, and it was also common to have another oscillation start in one and complete in the subsequent GLG. Occasionally, an oscillation spanned over two to three GLGs (e.g. PI-82-63, the 4^th^ δ^13^C oscillation starts in GLG 3 and completes in GLG 5), or no oscillation in some GLGs (e.g. PI-15-1091, missing δ^13^C oscillations in GLG 3 and 4), or multiple oscillations in one GLG (e.g. PI-17-1383, two complete δ^15^N oscillations in GLG 1; this most often occurred when five or more accessory layers were sampled). The magnitude of δ^15^N oscillations across the GLGs of 31 whales ranged from 0.2‰ to 3.4‰. The mean magnitude per whale ranged from 0.4‰ to 2.5‰ for δ^15^N (table 1). The magnitude of δ^13^C cycles for the 31 whales ranged from 0.1‰ to 2.6‰ across GLGs. The mean δ^13^C magnitude per whale varied from 0.2‰ to 1.1‰ (table 1).

**Table 1.** Magnitude of δ^15^N and δ^13^C oscillations in dentine accessory layers sampled along tooth growth layer groups (GLGs) in narwhals (*n* = 31, female (*n* = 17), male (*n* = 14)) hunted in 1982, 1983, 2015 and 2017 near Pond Inlet, Nunavut. Magnitude was calculated by subtracting the trough value from the peak value for each δ^15^N and δ^13^C oscillation (means and ranges are for each individual whale).

ID	sex	δ^13^C (‰) mean	δ^13^C (‰) range	δ^15^N (‰) mean	δ^15^N (‰) range
PI-83-02	F	0.4	0.2–0.7	1.4	0.5–2.2
PI-83-11	F	0.4	0.3–0.6	1.0	0.7–1.6
PI-83-13	F	0.3	0.1–0.4	1.5	0.4–2.2
PI-82-10	F	0.2	0.1–0.5	0.7	0.3–1.2
PI-82-11	F	0.2	0.1–0.3	0.6	0.4–0.9
PI-82-19	F	0.4	0.2–0.8	0.6	0.4–1.1
PI-82-31	F	0.3	0.1–1.5	0.8	0.4–1.5
PI-82-32	F	0.3	0.1–0.5	0.6	0.2–0.8
PI-82-13	M	0.5	0.2–1.0	0.7	0.3–0.9
PI-82-20	M	0.5	0.2–0.6	0.9	0.2–2.9
PI-82-21	M	0.4	0.2–0.6	0.4	0.2–0.8
PI-82-25	M	0.3	0.1–0.6	0.5	0.3–0.8
PI-82-28	M	0.3	0.2–0.6	0.7	0.5–1.0
PI-82-46	M	0.4	0.3–0.6	0.8	0.4–1.0
PI-82-63	M	0.3	0.2–0.5	0.7	0.4–1.3
PI-82-64	M	0.4	0.2–0.8	1.2	0.7–2.7
PI-82-65	M	0.5	0.2–1.4	1.8	1.3–2.8
PI-15-1039	F	0.6	0.2–1.0	0.4	0.2–0.9
PI-15-1053	F	0.4	0.2–0.9	0.5	0.3–0.8
PI-15-1054	F	0.5	0.3–0.8	0.6	0.2–1.1
PI-15-1056	F	0.4	0.1–0.7	0.6	0.3–1.0
PI-15-1057	F	0.7	0.4–1.5	2.2	1.9–2.6
PI-15-1058	F	0.5	0.2–0.9	0.6	0.4–1.1
PI-15-1089	F	1.1	0.4–2.6	2.5	2.1–3.0
PI-15-1090	F	0.4	0.2–0.6	2.0	0.6–2.6
PI-15-1091	F	0.5	0.1–1.2	0.8	0.3–1.3
PI-15-1041	M	0.7	0.2–2.3	1.7	1.1–2.2
PI-17-1363	M	0.4	0.2–0.7	0.4	0.3–0.9
PI-17-1380	M	0.5	0.2–0.9	1.3	0.5–2.0
PI-17-1383	M	0.4	0.1–1.0	2.4	1.7–3.4
PI-17-HK05	M	0.5	0.2–0.8	1.5	0.7–2.2

## 4. Discussion

Isotopic profiles in accessory layers within the narwhal GLGs revealed broad variability in intra-annual δ^15^N and δ^13^C patterns yet all exhibited cyclical oscillations. Although the oscillation frequencies were inconsistent, at least one oscillation per year was found in all whales, suggesting intra-annual variation in narwhal longitudinal δ^15^N and δ^13^C profiles. No sex-related differences in SI oscillations were observed, which was expected given that they have similar diets [43] and generally follow the same migration routes [16,23,43,44], and therefore are expected to integrate similar baseline SI variation. Although females tend to migrate slightly later than males (< 1 week) [23], this short time difference would not be relevant to the temporal scale we can evaluate in the GLG accessory layers in teeth. Overall, our findings agree with those of Nweeia *et al*. [35] who reported alternating high and low δ^15^N values along the dentine GLGs of a male narwhal tusk. Intuitively, the number of accessory layers we were able to sample impacted detectability of oscillations within a GLG (for example, multiple oscillations could not be detected within a GLG for which only two accessory layers could be sampled). However, it is unclear when the accessory layers are formed and how quickly they develop. While we are limited in our ability to interpret the observed SI patterns within a temporal context, oscillations within GLGs represent cyclical, intra-annual variation, representing seasonal variation in narwhal ecology (e.g. movement, diet) and/or physiology.

Narwhals migrate between inlets and fiords of the Canadian Arctic Archipelago and northern Baffin Bay in summer to central and southern Baffin Bay and Davis Strait in winter, however, the baseline SI across this range is not well defined. The baseline SI reported for the Baffin Bay area by Graham *et al*. [29] suggested no difference in δ^15^N or δ^13^C across the area. However, Pomerleau *et al*. [17] provided evidence of latitudinal gradients for both ẟ^15^N and ẟ^13^C in Baffin Bay, with higher ẟ^15^N of zooplankton species in narwhal summer areas (higher latitude) than in winter areas (lower latitude), and an inverse trend in ẟ^13^C. For example, the amphipod *Themisto libellula*, one of the most important prey species of adult Arctic cod, sampled in Baffin Bay and Davis Strait showed a 0.7‰ decrease in ẟ^15^N and 0.4‰ increase in ẟ^13^C [17] (figure 1). If SI variations along narwhal dentine were only influenced by baseline SI variations due to migration between summer and winter grounds, then we would expect to see either no changes assuming no latitudinal SI variations [29] or possibly inversely related cycles in ẟ^15^N and ẟ^13^C with magnitudes of approximately 0.7‰ and 0.4‰, respectively [17]. While the mean magnitude of approximately 50% of the whales was within this range (mean ẟ^15^N magnitude ≤ 0.7‰, *n* = 12; mean ẟ^13^C magnitude ≤ 0.4‰, *n* = 15), oscillations exceeding such magnitudes existed in the SI profiles of all narwhals. Furthermore, ẟ^15^N and ẟ^13^C profiles of most narwhals do not have an inverse relationship (figure 3). Therefore, although baseline differences between summer and winter habitats may contribute to small magnitude oscillations, they cannot explain the frequency and magnitude of the oscillations observed in our study.

Compared with the small baseline SI differences that narwhals may experience from migration, SI variations related to seasonal dietary shifts would be expected to have a stronger influence on the observed narwhal SI oscillations. Narwhals are known to feed intensively during winter in central/southern Baffin Bay and Davis Strait, where they build up body reserves on energy-rich Greenland halibut [19]. In summer, narwhals feed opportunistically and primarily on prey at lower trophic levels, such as Arctic cod [19]. The isotopic difference between Greenland halibut from Davis Strait and Arctic cod from the Central Canadian Arctic Archipelago can be up to 2.0‰ for ẟ^15^N and 0.5‰ for ẟ^13^C (figure 1, table 2) [20,43,46], which could drive a ẟ^15^N oscillation magnitude ˃1.0‰. In addition to seasonal dietary shifts, variation in prey composition of narwhals within and across years could also contribute to the large magnitude range for ẟ^15^N (0.2‰ to 3.4‰) and ẟ^13^C (0.1‰ to 2.6‰) oscillations within and among narwhals, as the isotopic differences among prey items can vary from zero to approximately 5.0‰ for ẟ^15^N and zero to approximately 2.0‰ for ẟ^13^C (table 2).

**Table 2.** Common narwhal prey δ^15^N and δ^13^C, along with sampling location and year, from previous studies.

				δ^15^N		δ^13^C		
**name**	**species**	**location collected**	***n***	**mean**	***SD***	**mean**	***SD***	**reference**
Arctic cod	*Boregadus saida*	Resolute Passage	26	15.2	0.7	−18.9	1.0	[45]
		Davis Strait	2	15.8	0.3	−19.5	0.1	[43]
		Resolute Bay	200	14.6	0.7	−19.4	0.7	[46]
		Davis Strait	15	14.0	1.0	−20.5	0.4	[20]
Greenland halibut	*Reinhardtius hippoglossoides*	Davis Strait	8	16.6	0.4	−19.9	0.6	[43]
		Davis Strait	20	15.0	1.7	−20.0	0.8	[20]
Capelin	*Mallotus villosus*	West Greenland	12	11.6	1.2	−19.0	0.6	[47]
Shrimp	*Pandalus borealis*	Clyde River	10	15.8	0.3	−19	0.2	[48]
		Davis Strait	10	14.5	0.5	−18.7	0.2	[20]
Squid	*Gonatus fabricii*	Davis Strait	2	16.0	0.4	−20.1	0.7	[43]
		Davis Strait	5	11.2	1.3	−19.8	0.6	[43]

Physiological factors related to nutrition (feeding intensity and efficiency), growth and reproduction can also affect δ^15^N and δ^13^C [26,49,50]. Narwhals are thought to feed intensively in winter to sustain year-round activities [19,51]. Catabolism of endogenous body reserves in summer could increase δ^15^N [52]. Alternatively, δ^15^N values may decrease if narwhals store enough body reserves or feed enough opportunistically in the summer to spare body protein catabolism while minimizing excretion of nitrogen waste (e.g. ^15^N-depleted urine) [53]. Unfortunately, not enough is known about the narwhal physiology and seasonal nutritional stress to tease apart these two possibilities, but isotopic oscillation due to such physiological factors may play a role in the observed oscillations.

Growth and reproduction can also affect δ^15^N and δ^13^C values. The rapid growth of narwhals between age 1 and 11 [54,55] may cause lower diet-tissue fractionation and thus lower δ^15^N [56,57], but this would not lead to isotopic oscillations within and across years. Similarly, gestation and lactation can result in changes in female δ^15^N and δ^13^C [58–60], but this would not oscillate within and across years as narwhals have ˃ 12 month gestation and lactation periods [31,61]. The physiology of breeding narwhal males is very poorly understood; however, if we assume that breeding males have higher energy expenditure and experience greater nutritional stress during the breeding season, then oscillations in δ^15^N could be expected for breeding male narwhals [26]. Regardless, all GLGs [1–11] sampled for whales in this study represent a period where narwhals are not yet sexually mature [55], hence reproduction overall is less likely to be an important factor for the SI oscillations observed in our study.

Interestingly, the narwhals in our study did not display oscillations with consistent patterns in their SI profiles. Although more SI fluctuations were generally observed in GLGs with more accessory layers (e.g. PI-17-1383, two δ^15^N oscillations in GLG 1 with eight accessory layers), more accessory layers did not necessarily result in more SI oscillations (e.g. PI-17-1380*,* one peak and one trough in GLG 3 with five accessory layers versus two peaks and one trough in GLG 6 with three accessory layers). In some cases, the oscillation spanned multiple GLGs or was absent in some GLGs. The most straightforward explanation for these SI observations is that the narwhal diet or distribution did not vary seasonally in those years. If narwhals shift their diet between isotopically similar prey in different areas, then such shifts would not cause SI oscillations. Alternative migration routes are another possibility: although narwhals do not remain in Eclipse Sound year-round due to sea ice, it is possible (although not likely) that narwhals could overwinter in the neighbouring Northwater polynya or move southwest (one narwhal migrated through Prince Regent Inlet to Northern Foxe Basin [62]), where prey and baseline δ^15^N and δ^13^C might be isotopically similar (but not likely since these are very different ecosystems) so as to dampen or eliminate SI oscillations.

Accessory layer is also subjective and limited by the GLG width. Periods of low food consumption can lead to thinner or no accessory layers [61,63], so SI fluctuations reflecting resource scarcity could be underrepresented. GLGs and accessory layers can also be difficult to discern, making it challenging to accurately assign accessory layers to their respective GLGs. We acknowledge that this source of error may have sometimes influenced patterns within a given year (i.e. due to erroneous assignment of an accessory layer from the previous or subsequent GLG), but not to the extent that the overall seasonal patterns evident across our data were obscured.

More research is needed to confirm and separate the underlying sources contributing to isotopic oscillations in narwhal embedded canines, but they provide a unique, fine-resolution longitudinal record of the narwhal life history. Better prey and regional baseline δ^15^N and δ^13^C data would help constrain diet versus distribution interpretations of SI cycles, as would improved knowledge of physiological factors that influence SI fractionation (e.g. seasonal energetics, reproductive state) and accessory layer formation. Overall, the intra-annual SI oscillations revealed in this study suggest variability in narwhal ecology and/or physiology related to environmental variation (i.e. seasonality), yet it is unclear to what extent such ecological or physiological variation may enhance narwhals’ resiliency or even adaptability to longer term, unidirectional climate change.

## Data Availability

Data are submitted as supplementary material and will be made available on Polar Data Catalogue [64]. Supplementary material is available online [65].

## References

[1] Barboza PS, Parker KL. 2008 Allocating protein to reproduction in Arctic reindeer and caribou. Physiol. Biochem. Zool. **82**, 835–855. (10.1086/596326) 18702605

[2] Williams T, Noren S, Glenn M. 2011 Extreme physiological adaptations as predictors of climate-change sensitivity in the narwhal, Monodon monoceros. Mar. Mammal Sci. **27**, 334–349. (10.1111/j.1748-7692.2010.00408.x)

[3] Harington CR. 2008 The evolution of Arctic marine mammals. Ecol. Appl. **18**, S23–S40. (10.1890/06-0624.1) 18494361

[4] Blix AS. 2016 Adaptations to polar life in mammals and birds. J. Exp. Biol. **219**, 1093–1105. (10.1242/jeb.120477) 27103673

[5] Moore SE, Huntington HP. 2008 Arctic marine mammals and climate change: impacts and resilience. Ecol. Appl. **18**, S157–S165. (10.1890/06-0571.1) 18494369

[6] Laidre KL, Stirling I, Lowry LF, Wiig Ø, Heide-Jørgensen MP, Ferguson SH. 2008 Quantifying the sensitivity of Arctic marine mammals to climate-induced habitat change. Ecol. Appl. **18**, S97–S125. (10.1890/06-0546.1) 18494365

[7] Serreze MC, Stroeve J. 2015 Arctic sea ice trends, variability and implications for seasonal ice forecasting. Phil. Trans. R. Soc. A **373**, 20140159. (10.1098/rsta.2014.0159) 26032315 PMC4455712

[8] Li WKW, McLaughlin FA, Lovejoy C, Carmack EC. 2009 Smallest algae thrive as the Arctic ocean freshens. Science **326**, 539–539. (10.1126/science.1179798) 19900890

[9] Blais M, Ardyna M, Gosselin M, Dumont D, Bélanger S, Tremblay J, Gratton Y, Marchese C, Poulin M. 2017 Contrasting interannual changes in phytoplankton productivity and community structure in the coastal Canadian Arctic Ocean. Limnol. Oceanogr. **62**, 2480–2497. (10.1002/lno.10581)

[10] McNicholl DG, Davoren GK, Reist JD. 2018 Life history variation across latitudes: observations between capelin (Mallotus villosus) from Newfoundland and the eastern Canadian Arctic. Polar Biol. **41**, 643–651. (10.1007/s00300-017-2225-x)

[11] Vihtakari M, Hordoir R, Treble M, Bryan MD, Elvarsson B, Nogueira A, Hallfredsson EH, Christiansen JS, Albert OT. 2021 Pan-Arctic suitable habitat model for Greenland halibut. ICES J. Mar. Sci. **78**, 1340–1356. (10.1093/icesjms/fsab007)

[12] Hamilton CD, Kovacs KM, Ims RA, Aars J, Lydersen C. 2017 An Arctic predator–prey system in flux: climate change impacts on coastal space use by polar bears and ringed seals. J. Anim. Ecol. **86**, 1054–1064. (10.1111/1365-2656.12685) 28415134

[13] Hauser DDW, Laidre KL, Stafford KM, Stern HL, Suydam RS, Richard PR. 2017 Decadal shifts in autumn migration timing by Pacific Arctic beluga whales are related to delayed annual sea ice formation. Glob. Chang. Biol. **23**, 2206–2217. (10.1111/gcb.13564) 28001336

[14] Dietz R, Desforges JP, Rigét FF, Aubail A, Garde E, Ambus P, Drimmie R, Heide-Jørgensen MP, Sonne C. 2021 Analysis of narwhal tusks reveals lifelong feeding ecology and mercury exposure. Curr. Biol. **31**, 1–8. 2012–2019.(10.1016/j.cub.2021.02.018) 33705717

[15] Koski WR, Davis RA. 1994 Distribution and numbers of narwhals (Monodon moneros) in baffin bay davis strait. Bioscience **39**, 15–40.

[16] Heide‐Jørgensen MP, Richard PR, Dietz R, Laidre KL. 2013 A metapopulation model for Canadian and West Greenland narwhals. Anim. Conserv. **16**, 331–343. (10.1111/acv.12000)

[17] Pomerleau C, Winkler G, Sastri AR, Nelson RJ, Vagle S, Lesage V, Ferguson SH. 2011 Spatial patterns in zooplankton communities across the eastern Canadian sub-Arctic and Arctic waters: insights from stable carbon (δ^13^C) and nitrogen (δ^15^N) isotope ratios. J. Plankton Res. **33**, 1779–1792. (10.1093/plankt/fbr080)

[18] Finley KJ, Gibb EJ. 1982 Summer diet of the narwhal (Monodon monoceros) in pond inlet, Northern Baffin Island. Can. J. Zool. **60**, 3353–3363. (10.1139/z82-424)

[19] Laidre KL, Heide-Jorgensen MP. 2005 Winter feeding intensity of narwhals (Monodon monoceros). Mar. Mammal Sci. **21**, 45–57. (10.1111/j.1748-7692.2005.tb01207.x)

[20] Watt CA, Heide-Jørgensen MP, Ferguson SH. 2013 How adaptable are narwhal? A comparison of foraging patterns among the world’s three narwhal populations. Ecosphere **4**, 71. (10.1890/es13-00137.1)

[21] Baffinland. 2018 Mary river project, phase 2 proposal, final impact assessment statement. Baffinland iron mines. NIRB file #08MN053.

[22] Lefort KJ, Garroway CJ, Ferguson SH. 2020 Killer whale abundance and predicted narwhal consumption in the Canadian Arctic. Glob. Chang. Biol. **26**, 4276–4283. (10.1111/gcb.15152) 32386346

[23] Shuert CR, Marcoux M, Hussey NE, Heide-Jørgensen MP, Dietz R, Auger-Méthé M. 2022 Decadal migration phenology of a long-lived Arctic icon keeps pace with climate change. Proc. Natl Acad. Sci. USA **119**, e2121092119. (10.1073/pnas.2121092119) 36279424 PMC9659343

[24] DeNiro M, Epstein S. 1978 Influence of diet on the distribution of carbon isotopes in animals. Geochim. Et Cosmochim. Acta **42**, 495–506.

[25] Bearhop S, Adams CE, Waldron S, Fuller RA, Macleod H. 2004 Determining trophic niche width: a novel approach using stable isotope analysis. J. Anim. Ecol. **73**, 1007–1012. (10.1111/j.0021-8790.2004.00861.x)

[26] Newsome SD, Clementz MT, Koch PL. 2010 Using stable isotope biogeochemistry to study marine mammal ecology. Mar. Mammal Sci. 509–572 (10.1111/j.1748-7692.2009.00354.x)

[27] Scheffer VB. 1950 Growth layers on the teeth of pinnipedia as an indication of age. Science **112**, 309–311. (10.1126/science.112.2907.309.b) 14781740

[28] Hobson KA, Sease JL. 1998 Stable isotope analyses of tooth annuli reveal temporal dietary records: an example using steller sea lions. Mar. Mammal Sci. **14**, 116–129. (10.1111/j.1748-7692.1998.tb00694.x)

[29] Graham BS, Koch PL, Newsome SD, McMahon KW, Aurioles D. 2010 Using isoscapes to trace the movements and foraging behavior of top predators in oceanic ecosystems. In Isoscapes (eds G Bowen, K Tu), pp. 299–318. Dordrecht, Netherlands: Springer. (10.1007/978-90-481-3354-3)

[30] Matthews CJD, Ferguson SH. 2014 Spatial segregation and similar trophic-level diet among eastern Canadian Arctic/north-west Atlantic killer whales inferred from bulk and compound-specific isotopic analysis. J. Mar. Biol. Assoc. U. K. **94**, 1343–1355.

[31] Zhao ST, Matthews CJD, Davoren GK, Ferguson SH, Watt CA. 2021 Ontogenetic profiles of dentine isotopes (δ^15^N and δ^13^C) reveal variable narwhal Monodon monoceros nursing duration. Mar. Ecol. Prog. Ser. **668**, 163–175. (10.3354/meps13738)

[32] Hay KA. 1980 Age determination of the narwhal, Monodon monoceros L. Rep. Int.l Whal.Commn 119–132.

[33] Watt CA, Stewart BE, Loseto L, Halldorson T, Ferguson SH. 2020 Estimating narwhal (Monodon monoceros) age using tooth layers and aspartic acid racemization of eye lens nuclei. Mar. Mammal Sci. **36**, 103–115. (10.1111/mms.12623)

[34] Rountrey AN, Fisher DC, Vartanyan S, Fox DL. 2007 Carbon and nitrogen isotope analyses of a juvenile woolly mammoth tusk: evidence of weaning. Quat. Int. **169**, 166–173. (10.1016/j.quaint.2006.08.002)

[35] Nweeia M, Thackeray J, Eichmiller F, Richard P, Leclerc LM, Lanham J, Newton I. 2008 A note on isotopic analysis of sectioned tusks of the narwhal (Monodon monoceros) and tusk growth rates. Ann. Transvaal Mus. **45**, 138–142.

[36] Feyrer LJ, Zhao ST, Whitehead H, Matthews CJD. 2020 Prolonged maternal investment in northern bottlenose whales alters our understanding of beaked whale reproductive life history. PLoS One **15**, e0235114. (10.1371/journal.pone.0235114) 32574188 PMC7310684

[37] Brault EK, Koch PL, Gier E, Ruiz‐Cooley RI, Zupcic J, Gilbert KN, McCarthy MD. 2014 Effects of decalcification on bulk and compound‐specific nitrogen and carbon isotope analyses of dentin. Rapid Commun. Mass Spectrom. **28**, 2744–2752. (10.1002/rcm.7073) 25380497

[38] Matthews CJD, Ferguson SH. 2014 Validation of dentine deposition rates in beluga whales by interspecies cross dating of temporal δ13c trends in teeth. vol. 10. The North Atlantic Marine Mammal Commission Scientific Publications. (10.7557/3.3196)

[39] Cowpertwait P, Metcalfe A. 2009 Introductory time series with R. New York, NY: Springer.

[40] Klvana I, Berteaux D, Cazelles B. 2004 Porcupine feeding scars and climatic data show ecosystem effects of the solar cycle. Am. Nat. **164**, 283–297. (10.1086/423431) 15478085

[41] Matthews CJD, Ferguson SH. 2015 Seasonal foraging behaviour of Eastern Canada-West Greenland bowhead whales: an assessment of isotopic cycles along baleen. Mar. Ecol. Prog. Ser. **522**, 269–286. (10.3354/meps11145)

[42] R Core Team. 2024 R: a language and environment for statistical computing. R Foundation for Statistical Computing.

[43] Watt CA, Ferguson SH. 2015 Fatty acids and stable isotopes (δ^13^C and δ^15^N) reveal temporal changes in narwhal (Monodon monoceros) diet linked to migration patterns. Mar. Mammal Sci **31**, 21–44. (10.1111/mms.12131)

[44] Watt CA, Orr JR, Ferguson SH. 2017 Spatial distribution of narwhal (Monodon monoceros) diving for Canadian populations helps identify important seasonal foraging areas. Can. J. Zool. **95**, 41–50. (10.1139/cjz-2016-0178)

[45] Hobson K, Welch H. 1992 Determination of trophic relationships within a high Arctic marine food web using δ^13^C and δ^15^N analysis. Mar. Ecol. Prog. Ser. **84**, 9–18. (10.3354/meps084009)

[46] Landry JJ, Fisk AT, Yurkowski DJ, Hussey NE, Dick T, Crawford RE, Kessel ST. 2018 Feeding ecology of a common benthic fish, shorthorn sculpin (Myoxocephalus scorpius) in the high arctic. Polar Biol. **41**, 2091–2102. (10.1007/s00300-018-2348-8)

[47] Møller P. 2006 Lipids and stable isotopes in marine food webs in West Greenland. In Dissertation. Lyngby, Denmark: Technical University of Denmark.

[48] Pedro S, Fisk AT, Ferguson SH, Hussey NE, Kessel ST, McKinney MA. 2020 Broad feeding niches of capelin and sand lance may overlap those of polar cod and other native fish in the Eastern Canadian Arctic. Polar Biol. **43**, 1707–1724. (10.1007/s00300-020-02738-8)

[49] Martínez del Rio C, Wolf N, Carleton SA, Gannes LZ. 2009 Isotopic ecology ten years after a call for more laboratory experiments. Biol. Rev. **84**, 91–111. (10.1111/j.1469-185x.2008.00064.x) 19046398

[50] Teixeira CR, Troina GC, Daura‐Jorge FG, Simões‐Lopes PC, Botta S. 2022 A practical guide on stable isotope analysis for cetacean research. Mar. Mammal Sci. **38**, 1200–1228. (10.1111/mms.12911)

[51] Chambault P, Blackwell SB, Heide-Jørgensen MP. 2023 Extremely low seasonal prey capture efficiency in a deep-diving whale, the narwhal. Biol. Lett. **19**, 20220423. (10.1098/rsbl.2022.0423) 36974666 PMC9943871

[52] Cherel Y, Hobson KA, Bailleul F, Groscolas R. 2005 Nutrition, physiology, and stable isotopes: new information from fasting and molting penguins. Ecology **86**, 2881–2888. (10.1890/05-0562)

[53] Aguilar A, Giménez J, Gómez–Campos E, Cardona L, Borrell A. 2014 δ^15^N value does not reflect fasting in mysticetes. PLoS One **9**, e92288. (10.1371/journal.pone.0092288) 24651388 PMC3961314

[54] Hay K. 1984 The life history of the narwhal (Monodon monoceros L.) in the Eastern Canadian Arctic. PhD dissertation, [Montreal]: McGill University.

[55] Garde E, Hansen SH, Ditlevsen S, Tvermosegaard KB, Hansen J, Harding KC, Heide-Jørgensen MP. 2015 Life history parameters of narwhals (Monodon monoceros) from Greenland. J. Mammal. **96**, 866–879. (10.1093/jmammal/gyv110)

[56] Fry B, Arnold C. 1982 Rapid ^13^C/^12^C turnover during growth of brown shrimp (Penaeus aztecus). Oecologia **54**, 200–204. (10.1007/bf00378393) 28311429

[57] Martínez del Rio C, Wolf B. 2005 Mass-balance models for animal isotopic ecology. In Physiological and ecological adaptations to feeding in vertebrates (eds J Starck, T Wang), pp. 141–174. Enfield, New Hampshire: Science Publishers.

[58] Kelly JF. 2000 Stable isotopes of carbon and nitrogen in the study of avian and mammalian trophic ecology. Can. J. Zool. **78**, 1–27. (10.1139/cjz-78-1-1)

[59] Kurle CM, Worthy GAJ. 2001 Stable isotope assessment of temporal and geographic differences in feeding ecology of northern fur seals (Callorhinus ursinus) and their prey. Oecologia **126**, 254–265. (10.1007/s004420000518) 28547625

[60] Clark CT *et al*. 2016 Heavy with child? Pregnancy status and stable isotope ratios as determined from biopsies of humpback whales. Conserv. Physiol. **4**, 1–13. (10.1093/conphys/cow050) 27766149 PMC5070529

[61] Uno KT, Fisher DC, Schuster G, Wittemyer G, Douglas-Hamilton I, Omondi P, Litoroh M, Cerling TE. 2020 High-resolution stable isotope profiles of modern elephant (Loxodonta africana) tusk dentin and tail hair from Kenya: implications for identifying seasonal variability in climate, ecology, and diet in ancient proboscideans. Palaeogeogr. Palaeoclimatol. Palaeoecol. **559**, 109962. (10.1016/j.palaeo.2020.109962)

[62] Watt CA, Orr J, LeBlanc B, Richard P, Ferguson SH. 2012 Satellite tracking of narwhals (Monodon monoceros) from admiralty Inlet (2009) and eclipse sound (2010-2011). DFO Can. Sci. Advis. Sec. Res. Doc 17.

[63] Heredia FM, Sosa Drouville A, Srur AM, Crespo EA, Grandi MF. 2022 Climate anomalies influence tooth growth patterns of South American sea lions. Mar. Mammal Sci. **38**, 175–189. (10.1111/mms.12850)

[64] Zhao ST, Matthews C, Watt C. 2025. ẟ^15^N and ẟ^13^C Cycles in Narwhal (Monodon Monoceros) Embedded Teeth Reveal Seasonal Variation in Ecology and/or Physiology (10.21963/13388) PMC11896694 40078917

[65] Zhao ST, Matthews C, Watt CA. 2025 Supplementary material from: δ^15^N and δ^13^C cycles in narwhal (Monodon monoceros) embedded teeth reveal seasonal variation in ecology and/or physiology. FigShare (10.6084/m9.figshare.c.7700093) PMC11896694 40078917

